# Factors influencing HPV vaccination intention and uptake behaviors among medical students based on the health belief model and vaccine hesitancy theory: a cross-sectional study

**DOI:** 10.3389/fpubh.2026.1894458

**Published:** 2026-07-09

**Authors:** Hao Wang, Shiqi Zhao, Hua Lin, Siyu Zhao, Dehui Yin

**Affiliations:** School of Public Health, Xuzhou Medical University, Xuzhou, China

**Keywords:** health belief model, HPV vaccine, mediation analysis, medical students, vaccination intention

## Abstract

**Background:**

HPV infection is the leading cause of cervical cancer. HPV vaccination coverage among young people in China remains low. Medical students, as a high-risk group and future health educators, are critical for cervical cancer control.

**Methods:**

A questionnaire survey was conducted among 1,244 medical students from April to May 2026. Data were analyzed using SPSS 26.0, including *t*-test, correlation, regression, and serial mediation analysis (PROCESS Model 6).

**Results:**

Among 1,203 valid participants (51.0% male, 49.0% female, 71.8% having received HPV vaccines), HPV knowledge was positively correlated with both vaccination intention and actual vaccination behavior. The serial chain mediation analysis specifically focused on vaccination intention: HPV knowledge showed a significant direct associational link with vaccination intention (*β* = 0.1238, *p* < 0.001). Perceived susceptibility and perceived severity served as significant serial mediators for intention.

**Conclusion:**

HPV knowledge is correlated with vaccination intention via direct associations and serial mediating associations of perceived susceptibility and perceived severity. Based on these cross-sectional correlational findings, interventions targeting HPV knowledge improvement may correlate with elevated risk perception and stronger vaccination intention.

## 1 Introduction

Human papillomavirus (HPV) infection is one of the most common sexually transmitted diseases globally and is closely associated with the development of various diseases (1–3). Epidemiological studies have confirmed that persistent infection with high-risk HPV is the primary cause of cervical cancer and its precancerous lesions (4). Hundreds of thousands of women worldwide die from cervical cancer each year (5), imposing a substantial disease burden. Concurrently, low-risk HPV infection can lead to benign lesions such as genital warts (6). While not fatal, these severely impact patients’ quality of life and psychological well-being. Consequently, cervical cancer is recognized as a malignant tumor with a clear etiology that is both preventable and controllable.

The introduction of HPV vaccines has provided a breakthrough tool for cervical cancer prevention (7–9). Extensive evidence-based medical research demonstrates that HPV vaccines exhibit high effectiveness and good safety in preventing infections by vaccine-covered HPV types, as well as related genital warts and cervical lesions (7, 10–14). However, achieving ideal public health objectives relies on high levels of immunization coverage. Regrettably, despite China having incorporated HPV vaccines into immunization program pilots in some regions, the overall vaccination rate, especially among adolescents and young adults, remains significantly lower than that in developed countries (11, 15, 16). Young people, being in a sexually active period, constitute a high-risk age group for HPV infection (17); however, their insufficient proactive vaccination willingness and actual vaccination behavior have become a prominent shortcoming in prevention and control efforts (18, 19). Of particular concern is that groups with relatively high cognitive levels, such as university students, also commonly exhibit phenomena where vaccine awareness is not low but vaccination rates are not high, and doubts persist regarding vaccine safety and necessity (18, 20). This suggests that mere knowledge dissemination is not sufficient to translate into effective health actions. Among various youth groups, medical students hold a unique dual identity and exemplary significance. On one hand, they are in the high-risk age group for HPV infection, making them direct beneficiaries of vaccine protection (21). On the other hand, medical students represent the core force of the future healthcare sector, bearing multiple responsibilities including disease prevention, health education, and clinical diagnosis and treatment. Their knowledge, attitudes, and vaccination behaviors regarding the HPV vaccine not only directly relate to their own health protection but will also profoundly influence their professional confidence and willingness to recommend prophylactic vaccination to the public in the future (22). Medical students represent a critical “inflection point” in public health. Unlike the general student population, medical students are exposed to clinical risk environments earlier, making their susceptibility higher. More importantly, they serve as pre-professionals whose vaccine hesitancy could ripple outward; an unvaccinated or hesitant medical student today is highly likely to become a clinician who fails to recommend the HPV vaccine to patients tomorrow, multiplying vaccine hesitancy in the general public. Thus, isolating this specific cohort allows for interventions that protect both individual and future population health. Should medical students exhibit misconceptions or low vaccination rates concerning the HPV vaccine, they would not only miss a crucial opportunity to protect their own health but might also be unable to fulfill the role of professional, proactive health advocates in their future positions, thereby undermining the social credibility of vaccine promotion.

Therefore, focusing on medical students as a key population that can serve as a role model and exert influence, and thoroughly investigating the mechanisms of HPV vaccine hesitancy formation and strategies for promoting vaccination behavior among them, hold significant practical urgency and theoretical value for improving the overall vaccination rate among young people, strengthening the professional competence of public health professionals, and accelerating the achievement of cervical cancer elimination goals. This study aims to apply the Health Belief Model and vaccine hesitancy theory, taking medical students from Xuzhou Medical University as the study subjects. It will employ a questionnaire survey method to collect data on HPV-related knowledge, health beliefs, vaccine hesitancy, vaccination intention, and vaccination behavior. Statistical methods will be used to analyze the influencing factors of HPV vaccine vaccination intention and behavior among medical students. Furthermore, a chain mediation model will be utilized to reveal the intrinsic pathways of HPV knowledge, perceived susceptibility, and perceived severity on vaccination intention, thereby identifying the key constraining factors and mechanisms influencing HPV vaccination among medical students. This will provide empirical evidence and theoretical reference for developing targeted campus health intervention strategies, increasing HPV vaccination rates among medical students, leveraging medical students’ role as health exemplars, and assisting in achieving China’s cervical cancer prevention and elimination goals.

In this study, while the full HBM and VHS scales were used to explore overall correlations and correlates in the regression models, the serial mediation analysis specifically focused on the cognitive-perceptual pathway (Knowledge → Perceived Susceptibility → Perceived Severity). According to HBM, knowledge acts as a modifying factor that alters individual cognitive perceptions, and risk appraisal (susceptibility and severity) serves as the primary cognitive psychological prerequisite before an individual evaluates benefits and barriers. Other constructs, such as perceived barriers and vaccine concerns, were retained as independent covariates in the broader regression models rather than parallel mediators to avoid excessive model complexity and potential multi-collinearity.

Even among medical students with a medical background, previous studies have reported a generally low vaccination rate, whereas the samples in this study exhibited a higher level of vaccination. The relevant differences will be analyzed in the limitations section.

## 2 Materials and methods

### 2.1 Data source

From April to May 2026, undergraduate and graduate schools of Xuzhou Medical University were selected as the cluster sampling population. A total of 1,244 electronic questionnaires were distributed and collected in April 2026. Questionnaires with more than two missing entries are considered invalid and will be directly excluded. A small number of individual missing entries will not be included in statistical analysis. After excluding invalid questionnaires with incomplete basic information or missing content, 1,203 valid questionnaires were recovered, yielding an effective response rate of 96.70%.

This study adopted a cross-sectional design. All variables were measured at a single time point, so this study can only explore the correlation and mediating association among variables, and cannot determine causal relationships or the temporal sequence of variable changes. This study employed stratified cluster sampling. Initially, stratification was conducted based on grade levels (freshman to senior year, graduate students), followed by the random selection of entire classes as research clusters within each grade. The questionnaire was distributed through an online platform, relying on counselors from various majors at the school. Inclusion criteria: full-time students enrolled in medical and related majors at Xuzhou Medical University; voluntary participation in this study and signing of an informed consent form. Exclusion criteria: questionnaires with completion time less than 90 s, multiple-choice answers exhibiting obvious patterns of deceptive responses (such as selecting all the same options), or severe missing basic demographic information. Effective response rate = (Valid questionnaires / Total distributed questionnaires) × 100% = 96.70%.

Vaccination behavior is designated as a binary categorical variable: unvaccinated (1) and having received at least one dose of HPV vaccine (2) (including one dose, two doses, or the full series). Vaccination status is collected through self-reporting by participants. In this study, students who have received one or two doses of the multi-dose schedule are classified as the vaccinated group.

### 2.2 Research methods

#### 2.2.1 General demographic information

A self-designed general information questionnaire was used, covering items such as age, gender, grade, major, monthly household income.

#### 2.2.2 HPV knowledge questionnaire (HPV-KQ) (23)

The revised HPV Knowledge Questionnaire (HPV-KQ) was used, consisting of 13 items covering HPV transmission routes, disease risks, vaccine awareness, and related topics. Each item was answered with “Yes/No/Uncertain.” A correct answer received 1 point, while an incorrect or uncertain answer received 0 points. The total score ranged from 0 to 13, with higher scores indicating higher levels of HPV knowledge. The Cronbach’s *α* coefficient of the HPV-KQ scale was 0.87 (23). This scale has been validated by multiple domestic and international studies, demonstrating good reliability and validity.

#### 2.2.3 Health belief model scale (HBM) (24)

A scale measuring beliefs about HPV vaccination was constructed based on the Health Belief Model, comprising 4 dimensions and 14 items: (1) Perceived benefits (3 items); (2) Perceived susceptibility (2 items); (3) Perceived severity (4 items); (4) Perceived barriers (5 items). Each item was rated on a 4-point Likert scale (1 = strongly disagree, 4 = strongly agree). Dimension scores were calculated as total scores, with higher scores indicating stronger health beliefs. Higher scores for perceived benefits, susceptibility, and severity indicated stronger vaccination willingness, while higher scores for perceived barriers indicated lower vaccination willingness. This scale was adapted from widely used Health Belief Model research tools both domestically and internationally. The Cronbach’s *α* values for the four subscales ranged from 0.71 to 0.78 (24), indicating good construct validity and internal consistency.

#### 2.2.4 Vaccine hesitancy scale (VHS) (25)

The adapted Vaccine Hesitancy Scale was used, consisting of 2 dimensions and 10 items: (1) Vaccine concerns (5 items); (2) Perceived Inconvenience to vaccination (5 items). Each item was rated on a 5-point Likert scale (1 = strongly disagree, 5 = strongly agree). Higher total scores on the Perceived Inconvenience subdimension reflect stronger logistical obstacles to vaccination and greater vaccine hesitancy, which negatively predict HPV vaccination uptake. This scale was developed based on the global vaccine hesitancy report framework and has been widely applied in HPV vaccine-related research, demonstrating good reliability and validity. In this study, the Cronbach’s *α* coefficient of the scale was 0.919, with 0.851 for the “vaccine concerns” dimension and 0.866 for the “convenience/barriers to vaccination” dimension, indicating good internal consistency reliability.

#### 2.2.5 Vaccination willingness and vaccination behavior questionnaire

Vaccination behavior was measured as a binary variable: unvaccinated and having received at least one dose. Vaccination willingness was measured using two items assessing future vaccination likelihood and intention, rated on a 5-point Likert scale (1 = no intention at all, 5 = definitely get vaccinated), with higher scores indicating stronger willingness.

### 2.3 Statistical analysis methods

Statistical analyses were performed using SPSS 26.0 software, with a significance level of *α* = 0.05 (two-tailed tests). For descriptive analysis, continuous data were expressed as mean ± standard deviation, and categorical data as frequencies and proportions (*n*/%). For reliability and validity testing, internal consistency reliability was assessed using Cronbach’s *α* coefficient, construct validity was examined through exploratory factor analysis, and common method bias was evaluated using Harman’s single-factor test. For univariate analysis, the *χ*^2^ test was used for between-group comparisons of categorical data, while independent samples t-tests or one-way analysis of variance (ANOVA) were used for continuous data. Pearson’s correlation test was used to explore linear correlations between HPV knowledge, health beliefs, vaccine hesitancy, and vaccination willingness. Binary logistic regression was applied to analyze factors associated with HPV vaccination uptake (binary outcome: ≥1 dose vaccinated vs. unvaccinated). For mechanism analysis, the PROCESS macro (Model 6) was employed to test the chain mediation effect, verifying the hypothesized pathway of “HPV knowledge → perceived susceptibility → perceived severity → vaccination willingness.”

Effect size evaluation criteria were adopted according to conventional social science standards: For independent samples *t*-test, Cohen’s *d*: *d* < 0.2 (negligible effect), 0.2 ≤ *d* < 0.5 (small effect), 0.5 ≤ *d* < 0.8 (moderate effect), *d* ≥ 0.8 (large effect). For Pearson correlation coefficient *r*: *r* < 0.3 (weak/small correlation), 0.3 ≤ *r* < 0.5 (moderate correlation), *r* ≥ 0.5 (strong/large correlation). For regression and mediation effects, standardized coefficients and 95% confidence intervals (95% CIs) were reported to reflect the magnitude and statistical stability of effects.

### 2.4 Exploratory factor analysis and construct validity

The factor structure of the questionnaire was examined using principal component analysis with Kaiser’s varimax rotation. The Kaiser-Meyer-Olkin (KMO) measure of sampling adequacy was 0.893, and Bartlett’s test of sphericity was significant (χ^2^ = 20802.744, *p* < 0.001), indicating that the data were highly suitable for factor analysis. A six-factor solution with eigenvalues greater than 1.0 emerged, accounting for 63.53% of the total variance. The first factor explained 21.98% of the variance, with the remaining five factors contributing 18.35, 10.43, 4.90, 4.21, and 3.66%, respectively. After rotation, the cumulative variance explained remained at 63.53%, with the six factors explaining 17.22, 17.16, 14.60, 5.93, 4.34, and 4.28% of the variance, respectively. Communalities of the items ranged from 0.422 to 0.741, with most items exceeding the recommended threshold of 0.60. Factor loadings for the retained items on their respective factors were all above 0.63 (e.g., Q10 = 0.657, Q11 = 0.657), indicating satisfactory convergent validity. No significant cross-loadings were observed, supporting the discriminant validity of the scale. Taken together, these findings provide adequate evidence of the construct validity of the instrument for use in the current study sample.

In this study, the Cronbach’s *α* coefficients for each scale are as follows: HPV-KQ (HPV Knowledge Questionnaire): *α* = 0.801; Health Belief Model Scale (HBM): overall *α* = 0.838; Perceived Benefits (*α* = 0.761), Perceived Susceptibility (*α* = 0.721), Perceived Severity (*α* = 0.758), Perceived Barriers (α = 0.853); Vaccine Hesitation Scale (VHS): overall *α* = 0.844. In the current sample, all scales exhibited good internal consistency reliability.

## 3 Results

### 3.1 Common method bias

To avoid common method bias, this study implemented ex-ante control by collecting anonymous responses. Ex-post, Harman’s single-factor test was conducted using unrotated exploratory factor analysis on the 26 items of the core variables. The results showed that four factors had eigenvalues greater than 1, and the variance explained by the first common factor was 30.04%, which is below the critical threshold of 40%. Therefore, there is no serious common method bias in this study. Harman’s single-factor test is a conventional method for common method bias testing, but it has certain limitations; more rigorous methods will be adopted in future research.

#### 3.1.1 Gender differences in vaccination status and willingness

Chi-square test showed that female students had a significantly higher HPV vaccination rate (76.1%, 449/590) compared to male students (67.9%, 416/613; χ^2^ = 10.12, *p* = 0.001, *φ* = 0.092, small effect size). Independent-samples t-test revealed that female students also reported significantly stronger vaccination willingness (mean = 4.12, SD = 0.89) than male students (mean = 3.78, SD = 0.95; *t* = 6.41, *p* < 0.001, Cohen’s d = 0.37, small-to-moderate effect size).

### 3.2 General characteristics of participants

A total of 1,203 respondents were included in this study, of whom 613 were male (51.0%) and 590 were female (49.0%). The majority were third-year students (39.0%), followed by second-year (23.8%) and fourth-year students (17.2%), with smaller proportions of first-year, fifth-year, and graduate students and above. Regarding major distribution, nursing accounted for the largest proportion (33.4%), followed by preventive medicine (25.8%), clinical medicine (21.4%), stomatology (13.8%), and other majors (5.7%). Monthly household income was predominantly in the range of 5,001–8,000 RMB (35.5%), followed by 8,001–12,000 RMB (25.9%), 3,000–5,000 RMB and above 12,000 RMB (each accounting for 18.6%), with the lowest proportion of respondents reporting a monthly income below 3,000 RMB (1.4%) (Table 1).

**Table 1:** General characteristics of participants (*n* = 1,203).

Variable	Category	Number (*n*)	Percentage (%)	Number of vaccinated individuals (%)
Gender	Male	613	51.0	416 (67.9)
	Female	590	49.0	449 (76.1)
Grade	Freshman	79	6.6	
	Sophomore	286	23.8	
	Junior	469	39.0	
	Senior	207	17.2	
	Fifth-year	42	3.5	
	Postgraduate or above	120	10.0	
Major	Nursing	402	33.4	
	Stomatology	166	13.8	
	Clinical medicine	257	21.4	
	Preventive medicine	310	25.8	
	Other	68	5.7	
Monthly household income (RMB)	<3,000	17	1.4	
	3,000–5,000	224	18.6	
	5,001–8,000	427	35.5	
	8,001–12,000	311	25.9	
	>12,000	224	18.6	

The vaccinated group is defined as participants who have received at least one dose of HPV vaccine.

### 3.3 Differential testing

Independent samples *t*-test results showed that participants who had received the HPV vaccine had significantly higher scores in HPV-related knowledge (*t* = −6.02, *p* < 0.001, *d* = −0.41), perceived susceptibility (*t* = −6.40, *p* < 0.001, *d* = −0.41), and perceived severity (*t* = −4.42, *p* < 0.001, *d* = −0.27) compared to unvaccinated participants, all with small effect sizes. Conversely, unvaccinated participants had significantly higher scores in perceived barriers (*t* = 4.55, *p* < 0.001, *d* = 0.26), vaccine concerns (*t* = 3.97, *p* < 0.001, *d* = 0.23), and perceived inconvenience of vaccination (*t* = 10.13, *p* < 0.001, *d* = 0.61), with the difference in Perceived inconvenience of vaccination reaching a moderate effect size. The difference in perceived benefits scores between the two groups was only marginally significant (*t* = −2.06, *p* = 0.040, *d* = −0.12), with a very small effect size (Tables 2, 3).

**Table 2:** Differences in scores between vaccinated and unvaccinated groups.

Dimension	Group	*N*	Mean	Standard deviation	Standard error mean
HPV knowledge total score	Unvaccinated	339	7.57	3.491	0.190
	Vaccinated	864	8.87	3.092	0.105
Perceived benefits	Unvaccinated	339	8.18	2.080	0.113
	Vaccinated	864	8.47	2.483	0.084
Perceived susceptibility	Unvaccinated	339	4.84	1.652	0.090
	Vaccinated	864	5.52	1.667	0.057
Perceived severity	Unvaccinated	339	11.20	2.556	0.139
	Vaccinated	864	11.95	2.848	0.097
Perceived barriers	Unvaccinated	339	12.81	3.100	0.168
	Vaccinated	864	11.80	4.240	0.144
Vaccine concerns	Unvaccinated	339	13.90	3.889	0.211
	Vaccinated	864	12.84	4.811	0.164
Perceived inconvenience	Unvaccinated	339	15.77	4.165	0.226
	Vaccinated	864	12.94	4.835	0.164

The vaccinated group is defined as participants who have received at least one dose of HPV vaccine.

**Table 3:** Differences between vaccinated and unvaccinated groups.

Variable	Levene’s sig.	*t* (adjusted)	*p*-value	Mean difference (vaccinated–unvaccinated)	95% CI
HPV knowledge total score	<0.001	−6.019	<0.001	−1.305	[−1.720, −0.890]
Perceived benefits	<0.001	−2.056	0.040	−0.290	[−0.570, −0.010]
Perceived susceptibility	0.986	−6.377	<0.001	−0.679	[−0.890, −0.470]
Perceived severity	0.037	−4.424	<0.001	−0.749	[−1.080, −0.420]
Perceived barriers	<0.001	4.550	<0.001	1.009	[0.570, 1.450]
Vaccine concerns	<0.001	3.972	<0.001	1.061	[0.540, 1.580]
Perceived inconvenience	<0.001	10.125	<0.001	2.832	[2.230, 3.420]

The vaccinated group is defined as participants who have received at least one dose of HPV vaccine.

### 3.4 Correlation analysis results

Pearson correlation analysis showed that HPV vaccination behavior was significantly positively correlated with HPV-related knowledge (*r* = 0.405, *p* < 0.001), perceived benefits (*r* = 0.370, *p* < 0.001), perceived susceptibility (*r* = 0.312, *p* < 0.001), and perceived severity (*r* = 0.355, *p* < 0.001). It was significantly negatively correlated with perceived barriers (*r* = −0.145, *p* < 0.001) and perceived inconvenience of vaccination (*r* = −0.134, *p* < 0.001), but showed no significant association with vaccine concerns (*r* = −0.053, *p* = 0.067). Moderate to high correlations were observed among the dimensions of health beliefs, with the highest correlations found between perceived barriers and vaccine concerns (*r* = 0.699, *p* < 0.001), as well as between perceived barriers and perceived inconvenience of vaccination (*r* = 0.639, *p* < 0.001), suggesting a strong intrinsic relationship among these three variables (Table 4).

**Table 4:** Pearson correlation analysis results.

Variable	Gender	Knowledge	Perceived benefits	Perceived susceptibility	Perceived severity	Perceived barriers	Vaccine concerns	Perceived inconvenience	HPV vaccinated
Gender	1	−0.066*	0.017	−0.066*	0.081**	−0.104**	0.019	0.015	0.042
Knowledge	−0.066*	1	0.374**	0.267**	0.284**	−0.296**	−0.286**	−0.295**	0.405**
Perceived benefits	0.017	0.374**	1	0.615**	0.654**	0.046	−0.065*	−0.073*	0.370**
Perceived susceptibility	−0.066*	0.267**	0.615**	1	0.623**	0.178**	0.101**	0.025	0.312**
Perceived severity	0.081**	0.284**	0.654**	0.623**	1	0.169**	0.053	−0.015	0.355**
Perceived barriers	−0.104**	−0.296**	0.046	0.178**	0.169**	1	0.699**	0.639**	−0.145**
Vaccine concerns	0.019	−0.286**	−0.065*	0.101**	0.053	0.699**	1	0.794**	−0.053
Perceived inconvenience	0.015	−0.295**	−0.073*	0.025	−0.015	0.639**	0.794**	1	−0.134**
HPV vaccinated	0.042	0.405**	0.370**	0.312**	0.355**	−0.145**	−0.053	−0.134**	1

*p < 0.05, **p < 0.01. All correlation coefficients are reported with 95% CIs; effect sizes were categorized based on Cohen’s criteria. The vaccinated group is defined as participants who have received at least one dose of HPV vaccine.

### 3.5 Regression analysis

Demographic variables including gender, grade, major and monthly household income were tested in preliminary regression models. After comparison, these demographic variables showed weak predictive effects on vaccination intention and behavior, and their inclusion would increase model complexity without improving explanatory power. Meanwhile, to avoid extra multicollinearity and keep the model focused on core theoretical variables derived from HBM and Vaccine Hesitancy Theory, we finally excluded these demographic covariates from the formal regression models. Relevant demographic differences are discussed separately in the discussion section.

#### 3.5.1 Predicting vaccination willingness

A multiple linear regression analysis was conducted with future HPV vaccination willingness among the unvaccinated population as the dependent variable, and HPV knowledge and dimensions of health beliefs as independent variables. The results showed that the overall regression model was significant (*F* = 64.041, *p* < 0.001), with an adjusted R^2^ of 0.269, indicating that the model explained 26.9% of the variance in future vaccination willingness.

Specifically, HPV knowledge level (*β* = 0.262, *p* < 0.001), perceived benefits (*β* = 0.138, *p* < 0.001), perceived susceptibility (*β* = 0.067, *p* = 0.049), perceived severity (*β* = 0.172, *p* < 0.001), and vaccine concerns (*β* = 0.258, *p* < 0.001) was significantly positively associated with future vaccination willingness. In contrast, perceived barriers (*β* = −0.229, *p* < 0.001) and perceived inconvenience of vaccination (*β* = −0.104, *p* = 0.013) was significantly negative predictively associated with future vaccination willingness. Among these, HPV knowledge and vaccine concerns had the largest absolute standardized regression coefficients, indicating they were the important correlates of influencing vaccination willingness (Table 5).

**Table 5:** Regression results for vaccination willingness.

Variable	B	SE	Beta	t	*p*	95% CI for beta
Constant	1.259	0.177	—	7.110	<0.001	—
HPV knowledge total score	0.097	0.011	0.262	9.138	<0.001	[0.206, 0.317]
Perceived benefits	0.070	0.018	0.138	3.810	<0.001	[0.067, 0.206]
Perceived susceptibility	0.048	0.024	0.067	1.967	0.049	[0.001, 0.132]
Perceived severity	0.074	0.015	0.172	4.819	<0.001	[0.103, 0.239]
Perceived barriers	−0.069	0.011	−0.229	−6.222	<0.001	[−0.291, −0.164]
Vaccine concerns	0.068	0.012	0.258	5.736	<0.001	[0.198, 0.315]
Perceived inconvenience	−0.026	0.010	−0.104	−2.477	0.013	[−0.170, −0.037]

The vaccinated group is defined as participants who have received at least one dose of HPV vaccine.

Collinearity diagnostics were performed prior to conducting the regression analyses. For the multiple linear regression model (Table 5), the Variance Inflation Factors (VIF) for all correlate variables ranged from 1.124 to 2.415, and all tolerance values were well above 0.10 (ranging from 0.414 to 0.890). Therefore, no serious multi-collinearity issues were detected in the final regression models.

The multiple linear regression model was statistically significant (*F* = 64.041, *p* < 0.001), with an adjusted *R*^2^ of 0.269, explaining 26.9% of the variance in vaccination willingness. All significant correlates exhibited small to moderate effect sizes. Among them, HPV knowledge (*β* = 0.262, 95% CI [0.206, 0.317]) and vaccine concerns (*β* = 0.258, 95% CI [0.198, 0.315]) had the largest standardized coefficients, serving as the primary influencing factors with moderate effect sizes. Perceived benefits (*β* = 0.138, 95% CI [0.067, 0.206]), perceived susceptibility (*β* = 0.067, 95% CI [0.001, 0.132]), perceived severity (*β* = 0.172, 95% CI [0.103, 0.239]), perceived barriers (*β* = −0.229, 95% CI [−0.291, −0.164]) and perceived inconvenience (*β* = −0.104, 95% CI [−0.170, −0.037]) showed small to moderate effect sizes.

#### 3.5.2 Predicting vaccination behavior

A binary logistic regression analysis was conducted with HPV vaccination status (vaccinated/unvaccinated) as the dependent variable and HPV knowledge and dimensions of health beliefs as independent variables. Where the vaccinated group was defined as having received at least one dose of HPV vaccine. The results showed that the overall model was significantly (χ^2^ = 192.095, *p* < 0.001), associated with a Nagelkerke R^2^ of 0.212, indicating that the model explained 21.2% of the variance in vaccination behavior, and the overall prediction accuracy was 76.8%.

Specifically, HPV knowledge level (OR = 1.091, *p* < 0.001), perceived susceptibility (OR = 1.421, *p* < 0.001), and vaccine concerns (OR = 1.137, *p* < 0.001) was significantly positively associated with HPV vaccination behavior. In contrast, perceived benefits (OR = 0.836, *p* < 0.001) and perceived inconvenience of vaccination (OR = 0.795, *p* < 0.001) had significant negative predictive effects on vaccination behavior. Perceived severity and perceived barriers had no significant association with HPV vaccination behavior (*p* > 0.05) (Table 6).

**Table 6:** Logistic regression results for vaccination behavior.

Variable	B	SE	Wald χ^2^	*p*	OR	95% CI
Constant	1.179	0.407	8.382	0.004	3.251	—
HPV knowledge total score	0.087	0.024	13.534	<0.001	1.091	[1.041, 1.143]
Perceived benefits	−0.179	0.045	15.774	<0.001	0.836	[0.765, 0.914]
Perceived susceptibility	0.351	0.060	34.071	<0.001	1.421	[1.263, 1.598]
Perceived severity	0.046	0.036	1.591	0.207	1.047	[0.976, 1.123]
Perceived barriers	−0.020	0.027	0.520	0.471	0.981	[0.931, 1.034]
Vaccine concerns	0.129	0.027	22.001	<0.001	1.137	[1.079, 1.199]
Perceived inconvenience	−0.229	0.026	80.704	<0.001	0.795	[0.756, 0.836]

The vaccinated group is defined as participants who have received at least one dose of HPV vaccine.

Notably, perceived benefits showed a negative predictive effect on actual vaccination behavior, and vaccine concerns positively predicted vaccination intention and behavior. We rechecked the questionnaire scoring rules, data coding and model specification, and confirmed no operational errors. This unexpected result may be related to the unique group characteristics of medical students.

In binary logistic regression, the odds ratios (OR) and corresponding 95% CIs reflected the magnitude of predictive effects. HPV knowledge (OR = 1.091, 95% CI [1.041, 1.143]), perceived susceptibility (OR = 1.421, 95% CI [1.263, 1.598]) and vaccine concerns (OR = 1.137, 95% CI [1.079, 1.199]) positively predicted vaccination behavior with small to moderate effect sizes. Perceived benefits (OR = 0.836, 95% CI [0.765, 0.914]) and Perceived inconvenience (OR = 0.795, 95% CI [0.756, 0.836]) acted as negative correlates, also with small to moderate effect sizes. Perceived severity and perceived barriers showed no statistically significant predictive effects.

### 3.6 Chain mediation effect

The chain mediation analysis results showed (Table 7) that HPV knowledge had a significant direct positive effect on vaccination intention (effect = 0.124, *p* < 0.001). Additionally, three significant indirect pathways were identified:

1. Single mediation pathway 1: HPV knowledge indirectly associated with vaccination willingness by enhancing individuals’ perceived susceptibility to HPV infection (effect = 0.013, 95% CI [0.006, 0.020]).
2. Single mediation pathway 2: HPV knowledge indirectly associated with vaccination willingness by enhancing individuals’ perception of the severity of HPV infection (effect = 0.008, 95% CI [0.004, 0.013]).
3. Chain mediation pathway: HPV knowledge indirectly associated with vaccination willingness through the chain transmission of “perceived susceptibility → perceived severity” (effect = 0.010, 95% CI [0.005, 0.014]).

**Table 7:** Chain mediation effect analysis results.

Effect type	Pathway	Effect	Boot SE	95% confidence interval
Direct Effect	HPV Knowledge → Vaccination Willingness	0.124	0.010	[0.105, 0.143]
Indirect Effect 1	HPV Knowledge → Perceived Susceptibility → Vaccination Willingness	0.013	0.004	[0.006, 0.020]
Indirect Effect 2	HPV Knowledge → Perceived Severity → Vaccination Willingness	0.008	0.002	[0.004, 0.013]
Indirect Effect 3	HPV Knowledge → Perceived Susceptibility → Perceived Severity → Vaccination Willingness	0.010	0.002	[0.005, 0.014]
Total Indirect Effect	Sum of all indirect pathways	0.030	0.004	[0.023, 0.039]

**Figure 1:**
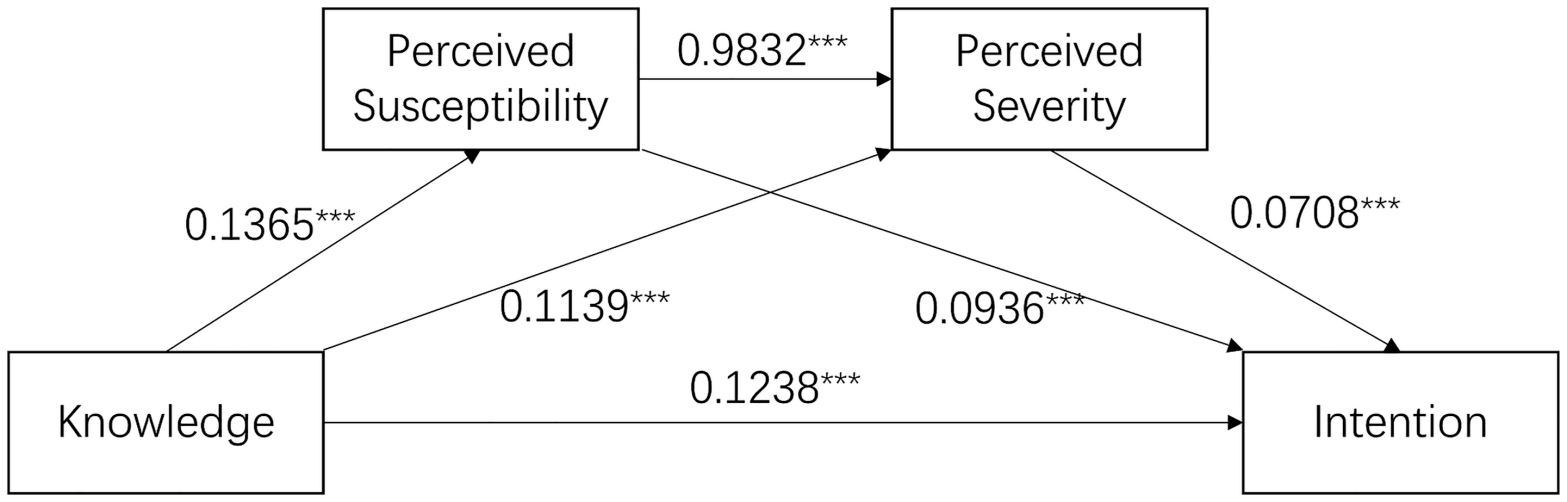
Mediating model diagram. ^***^p < 0.001, ^**^p < 0.01, ^*^p < 0.05.

The total association of the model was significant (effect = 0.030, 95% CI [0.023, 0.039]), indicating that HPV knowledge not only directly associated vaccination willingness but also indirectly associated vaccination willingness through the mediating role of health beliefs. The control variable gender also was associated with vaccination willingness (*β* = 0.410, *p* < 0.001) (see Figure 1; Table 7).

## 4 Discussion

Based on the Health Belief Model and Vaccine Hesitancy Theory, this study empirically analyzed the factors influencing HPV vaccination willingness and behavior among medical students. A chain mediation model was used to reveal the intrinsic pathway of “HPV knowledge → perceived susceptibility → perceived severity → vaccination willingness.” The results showed that HPV knowledge, health beliefs, vaccine concerns, and vaccination convenience collectively influenced vaccination willingness and behavior. Furthermore, perceived susceptibility and perceived severity played a significant chain mediating role in the effect of knowledge on vaccination willingness. The findings of this study provide a theoretical basis and practical reference for improving HPV vaccination rates among medical students and enhancing campus-based health intervention strategies. Notably, the overall HPV vaccination rate in our sample was higher than that reported in previous studies targeting general college students. This discrepancy can be explained by group specificity: as medical students possess professional medical knowledge and systematic health literacy, they have a more comprehensive understanding of HPV-related diseases and vaccines, which contributes to higher vaccination acceptance and actual uptake compared with non-medical university students.

First, HPV knowledge level among medical students is an important correlate of vaccination willingness and behavior. This study found that higher HPV knowledge scores were associated with stronger vaccination willingness and a higher vaccination rate, and knowledge directly and positively associated vaccination willingness. This finding is consistent with most domestic and international studies, suggesting that improving HPV-related knowledge remains a fundamental step in promoting vaccination. Although medical students have a certain medical professional background, issues such as incomplete knowledge and inaccurate understanding still exist, indicating that relying solely on professional coursework is insufficient to establish adequate awareness (26). Targeted and systematic health education is still needed. Incorporating topics such as HPV etiology, transmission routes, disease hazards, and vaccine efficacy into the routine education of medical students may be associated with their ability to make informed health decisions and lay a professional foundation for their future public health education efforts (27–29).

Second, health beliefs play a key role in HPV vaccination decision-making. This study showed that higher levels of perceived benefits, perceived susceptibility, and perceived severity were associated with more positive vaccination willingness and behavior, while higher perceived barriers were associated with lower vaccination willingness. These findings align with the core assumptions of the Health Belief Model. The vaccinated group had significantly higher scores for perceived susceptibility and perceived severity, indicating a greater awareness of their own infection risk and the harm of the disease. In contrast, the unvaccinated group had higher scores for perceived barriers, suggesting more prominent concerns about vaccination costs, side effects, and procedural inconveniences. These results suggest that intervention strategies should be advanced on two fronts: on the one hand, strengthening risk perception by emphasizing HPV exposure risk and the dangers of cervical cancer among young adults (30–32); on the other hand, reducing perceived barriers by alleviating excessive concerns about the vaccination process, cost, and safety, thereby increasing vaccination motivation (33).

Third, vaccine hesitancy and vaccination accessibility are associated with influencing vaccination behavior. This study showed that vaccine concerns was associated with both vaccination willingness and behavior, while higher perceived inconvenience of vaccination was associated with lower vaccination willingness and rates. These findings are generally consistent with Vaccine Hesitancy Theory. In the binary logistic regression, perceived inconvenience of vaccination was significantly negatively associated with vaccination behavior, suggesting that insufficient accessibility is a key barrier to vaccination. Busy academic schedules, time constraints, and lack of clarity regarding vaccination locations and procedures among medical students may reduce the likelihood of vaccination (34). Therefore, improving vaccination service accessibility, simplifying appointment processes, providing on-campus vaccination channels, and clarifying cost and safety information can be associated with vaccine hesitancy and enhance vaccination willingness (35).

Fourth, the chain mediation effect revealed the intrinsic pathway through which knowledge influences vaccination willingness. This study confirmed through Model 6 that HPV knowledge not only directly associated with vaccination willingness but also exerted a chain mediating effect through perceived susceptibility and perceived severity—namely, “increased knowledge → enhanced susceptibility → heightened severity perception → increased vaccination willingness.” This finding refines the transmission mechanism of the Health Belief Model: improved knowledge helps individuals more accurately assess the likelihood of infection, which in turn strengthens their judgment of disease harm, ultimately promoting vaccination willingness. Contextualized and individualized risk communication can strengthen beliefs (35), thereby achieving more stable and effective behavioral promotion outcomes (36, 37).

Based on the above findings, we propose a multi-component campus intervention strategy to improve HPV vaccination uptake among medical students. The integrated measures include: (1) targeted health education and personalized risk communication to elevate HPV-related knowledge and risk perception; (2) on-campus consultation services and vaccination reminders to reduce students’ doubts; (3) optimized digital appointment systems and transparent cost information to lower access barriers; (4) cooperating with campus clinics and nearby community pharmacies to provide on-site vaccination services. Accumulated evidence has proven that multi-dimensional, access-oriented interventions achieve better effects than single health education (38, 39).

Our findings highlight a clear intention-behavior discrepancy commonly termed the “intention-behavior gap” in health psychology. Although medical students showed relatively high willingness driven by knowledge and risk appraisal, actual uptake behavior was restricted by structural and convenience barriers (such as vaccine shortages or financial costs), as reflected by the strong correlation between barriers and vaccine hesitancy (*r* = 0.699). This gap underscores that improving cognitive awareness alone is insufficient; interventions must focus on removing logistic and financial barriers on campus.

Interestingly, in the multivariate regression models, several correlates exhibited directionalities opposite to their bivariate correlations. Specifically, vaccine concerns positively predicted willingness (*β* = 0.258) and behavior (OR = 1.137), while perceived benefits negatively predicted vaccination behavior (OR = 0.836). This phenomenon can be statistically attributed to a ‘suppression effect.’ Because vaccine concerns are heavily correlated with perceived barriers (*r* = 0.699) and perceived inconvenience (*r* = 0.794), when entered simultaneously into the regression model, the unique variance of vaccine concerns is suppressed, flipping its statistical sign. From a practical perspective, this suggests that among medical students, once systemic structural barriers (e.g., vaccine availability, financial costs) are controlled for, those who possess higher vaccine concerns might actually represent a group with greater health vigilance, driving them toward active vaccination behavior. Similarly, the negative coefficient of perceived benefits in logistic regression reflects that the variance shared with risk perceptions (susceptibility and severity) suppresses its independent main effect.

Additionally, this study found certain differences in demographic characteristics such as gender, grade, and major. Female students had relatively higher vaccination willingness, consistent with previous studies. As future healthcare providers, medical students’ vaccination attitudes and behaviors have a demonstration effect. Increasing the vaccination rate in this group not only protects individual health but also helps enhance their professional confidence in recommending vaccines to the public and improves the credibility of health education. Therefore, comprehensive HPV vaccination interventions targeting medical students have dual value: they are an important component of youth health promotion and a key element in strengthening the competencies of public health professionals.

Due to the cross-sectional design of this study, it is impossible to confirm causality. Although knowledge and beliefs have predictive power on willingness, future prospective intervention studies or longitudinal tracking studies are still needed to verify whether knowledge intervention can truly bridge the “willingness-behavior” gap and promote vaccination conversion.

## 5 Limitations

This study has the following limitations. First, this study employed a cross-sectional design, which can only reveal associations and mediating effects among variables but cannot infer strict causal relationships. Second, the study participants were from a single medical university, limiting the representativeness of the sample; therefore, generalization of the conclusions requires caution. Third, vaccination behavior relied on self-report, which may be subject to recall bias and social desirability bias. Future research should consider multicenter, prospective cohort studies to further validate causal relationships, along with empirical studies incorporating policy environments and campus-based interventions.

The HPV vaccination rate in this study sample is 71.8% (defined as having received at least one dose, including partial doses), which is significantly higher than the levels reported in previous studies on the general population and most medical students in China. This high rate exhibits notable sample specificity, and caution is needed when extrapolating to the national population. The reasons for this high vaccination rate include three points: ① During the same period of the survey, our university conducted HPV vaccine science popularization and on-campus vaccination special events, which improved students’ accessibility to vaccination; ② There is a response selection bias, as those who have completed the questionnaire are more likely to have participated in the vaccination, which may slightly overestimate the overall vaccination rate; ③ The subjects of this study are all medical students, who possess higher health literacy, are more likely to obtain vaccine-related medical information, and have better access to medical vaccination channels compared to the general youth population. In summary, the vaccination level of this sample cannot represent the overall vaccination status of ordinary college students and socially eligible women in China.

This study relies on self-reported data, which may introduce social desirability and self-report biases, potentially overestimating vaccination rates. Fourthly, although ex-post statistical checks minimized its threat, common method bias (CMB) cannot be entirely ruled out in a single-source questionnaire survey. Lastly, the high correlation between specific subscales indicates potential suppression effects in regression models, which calls for cautious interpretation of the individual predictive weights.

## 6 Conclusion

To conclude, multiple factors including HPV knowledge, health beliefs, vaccine concerns and vaccination convenience show significant correlations with medical students’ HPV vaccination willingness and actual uptake. This study observed an associational pathway where HPV knowledge relates to stronger vaccination willingness through perceived susceptibility and perceived severity. In light of the present results, we suggest developing multi-faceted interventions to improve HPV knowledge and risk perception, mitigate vaccination barriers and enhance service accessibility. Embedding HPV vaccine education into medical education systems may bring potential benefits to vaccination promotion. It should be noted that these recommendations are derived from cross-sectional data, and subsequent prospective studies are necessary to confirm their practical value.

## Data Availability

The original contributions presented in the study are included in the article/supplementary material, further inquiries can be directed to the corresponding author.
