# Correction: Factors influencing HPV vaccination intention and uptake behaviors among medical students based on the health belief model and vaccine hesitancy theory: a cross-sectional study

**DOI:** 10.3389/fpubh.2026.1940407

**Published:** 2026-07-31

**Authors:** Hao Wang, Shiqi Zhao, Hua Lin, Siyu Zhao, Dehui Yin

**Affiliations:** School of Public Health, Xuzhou Medical University, Xuzhou, China

**Keywords:** health belief model, HPV vaccine, mediation analysis, medical students, vaccination intention

**Affiliation** “School of Public Health, Xuzhou Medical University, Xuzhou, China” was erroneously written as “Graduate School, Xuzhou Medical University, Xuzhou, China.” This affiliation has now been updated for all authors.

The **Author contributions** statement was erroneously given as “HW: Writing – review & editing, Formal analysis, Supervision, Writing – original draft, Data curation, Software, Resources, Conceptualization, Funding acquisition, Validation, Project administration, Visualization, Investigation, Methodology. ShZ: Software, Investigation, Visualization, Writing – original draft, Data curation, Resources, Writing – review & editing, Validation, Funding acquisition, Methodology, Conceptualization, Formal analysis, Project administration, Supervision. HL: Validation, Project administration, Methodology, Data curation, Visualization, Conceptualization, Formal analysis, Writing – original draft, Writing – review & editing, Supervision, Funding acquisition, Resources, Software, Investigation. SiZ: Software, Investigation, Supervision, Funding acquisition, Conceptualization, Formal analysis, Visualization, Project administration, Writing – review & editing, Data curation, Resources, Methodology, Validation, Writing – original draft. DY: Data curation, Software, Methodology, Writing – original draft, Resources, Investigation, Visualization, Validation, Funding acquisition, Supervision, Formal analysis, Writing – review & editing, Conceptualization, Project administration.”

The correct **Author contributions** statement is “HW: Conceptualization, Investigation, Writing – review & editing. ShZ: Data curation, Writing – original draft, Writing – review & editing. HL: Formal analysis, Project administration, Writing – review & editing. SiZ: Validation, Writing – review & editing. DY: Writing – original draft, Writing – review & editing.”

Authors Hao Wang and Shiqi Zhao was erroneously unassigned as equal contributing authors sharing first authorship.

The original version of this article has been updated.

